# Improving the organization of palliative care: identification of barriers and facilitators in five European countries

**DOI:** 10.1186/s13012-014-0130-z

**Published:** 2014-10-16

**Authors:** Jasper van Riet Paap, Myrra Vernooij-Dassen, Frederike Brouwer, Franka Meiland, Steve Iliffe, Nathan Davies, Wojciech Leppert, Birgit Jaspers, Elena Mariani, Ragni Sommerbakk, Kris Vissers, Yvonne Engels, on behalf of the IMPACT research team

**Affiliations:** Scientific Institute for Quality of Healthcare (IQ healthcare), Radboud university medical center, P.O. Box 9101, 6500 HB Nijmegen, The Netherlands; Kalorama Foundation, Nijmegen, The Netherlands; Department of General Practice & Elderly Care Medicine, EMGO Institute for Health and Care Research, Alzheimer Center, VU University Medical Center, P.O. Box 7057, 1007 MB Amsterdam, The Netherlands; Department of Primary Care & Population Health, University College London, Royal Free Campus, Rowland Hill Street, London, NW3 2PF UK; Department of Palliative Medicine, Poznan University of Medical Sciences, 61-245, Poznan, Poland; Department of Palliative Medicine, University of Bonn, Sigmund-Freud-Str. 25, 53127 Bonn, Germany; Clinic for Palliative Medicine, University of Goettingen, Robert-Koch-Str. 40, 37075 Goettingen, Germany; Alma Mater Studiorum, Department of Psychology, University of Bologna, Bologna, Italy; European Palliative Care Research Centre, Department of Cancer Research and Molecular Medicine, Faculty of Medicine, Norwegian University of Science and Technology, P.O. Box 8905, NO-7491 Trondheim, Norway; Department of Anaesthesiology, Pain and Palliative Medicine, Radboud university medical center, P.O. Box 9101, 6500 HB Nijmegen, The Netherlands

**Keywords:** Palliative care, Barriers and facilitators, Quality improvement, Organization of care, Europe

## Abstract

**Background:**

Interventions to improve palliative care encounter challenges beyond the usual implementation problems because of palliative care’s complex and changing character. In this study, we explored barriers and facilitators faced by health-care professionals in five European countries (England, Germany, Italy, Norway and the Netherlands) with regard to improving the organization of their palliative care service.

**Methods:**

Semi-structured individual and focus group interviews were conducted with purposefully selected health-care professionals. The constant comparative method was used to analyse the data.

**Results:**

Professionals working in hospitals, hospices, nursing homes and primary care facilities who provide palliative care to adult patients were interviewed (*n* =40) or participated in ten focus group interviews (*n* =59). Barriers and facilitators were inductively grouped into 16 categories and arranged into five themes: innovation, individual professional level, group dynamics, organizational context and local political-economic context. Although the barriers and facilitators identified differed in scope, context, strength and provenance, they were shared by professionals from different European countries.

**Conclusion:**

This study identified barriers and facilitators to organizational change in palliative care. Some of these barriers and facilitators were experienced by professionals in almost all countries and are therefore prerequisites to change. Understanding the barriers to and facilitators of change will help tailor organizational improvements to the needs of individuals and organizations.

**Electronic supplementary material:**

The online version of this article (doi:10.1186/s13012-014-0130-z) contains supplementary material, which is available to authorized users.

## Background

Palliative care aims to preserve the best possible quality of life of the patient whose disease is not responsive to curative treatment [1]. Improvements in palliative care usually focus on pain and symptom control, use of standardized assessment tools, care in the last days of life and the quality of dying [2,3]. To date, improving specific organizational aspects of palliative care has received less attention [3].

Interventions to improve the organization of palliative care encounter challenges beyond the usual problems of implementation of change in health care. Patients in need of palliative care often move between services, have changing (and often increasing) needs for treatment and support, have multiple problems and symptoms [4] and receive care from a variety of professionals [5]. This requires optimal collaboration between patients, informal carers and a range of professionals and health-care organizations [1,6]. In order to overcome these challenges and improve the organization of palliative care, systematic implementation to translate the results of clinical research into everyday clinical routines is necessary [7]. A first step in a systematic implementation process is the identification of barriers and facilitators [7]. Recent studies have provided some insights in possible barriers and facilitators related to changing the organization of other fields in health care, for example, in the handover of care [8], case management [9] and the introduction of nursing guidelines [10]. However, studies on improving the organization of palliative care are still lacking.

For this reason, the objective of this study was to identify barriers to and facilitators of improvements in the organization of palliative care in Europe. The results from this study will be used in the European Seventh Framework IMPACT project (IMplementation of quality indicators for PAlliative Care sTudy) to develop and tailor national and setting-specific strategies to improve the organization of palliative care in England, Germany, Italy, Norway and the Netherlands [11].

## Methods

A qualitative design, with semi-structured individual and focus group interviews, was used. Individual interviews were conducted in order to gain professionals’ understanding of barriers and facilitators to improve the organization of palliative care [12]. Focus group interviews were used to reflect the social and cultural contexts of barriers and facilitators to improve the organization of palliative care [13].

### Participants and settings

The study took part in England, Germany, Italy, Norway and the Netherlands. Participants of the individual and focus group interviews were purposefully selected health-care professionals working in services providing palliative care. Besides professionals working in hospitals, hospices and primary care settings, also nursing home professionals were included because of the growing population in such settings in need of palliative care. Services which have been providing palliative care for adult patients for at least 2 years were eligible for this study. In each country, a snowballing method was used to select professionals for the individual and focus group interviews: all professionals approached were asked to nominate other professionals [14]. Professionals were included if they were either clinically involved in palliative care (e.g. nurses and physicians) or in the organization of palliative care (e.g. managers of a specific palliative care service) and if they had at least 1-year professional experience in palliative care. Recruitment continued until no new themes or information was coming out of the interviews.

### Data collection

A semi-structured interview guide, based on the literature and previous experiences of the research team, was developed and used to guide both individual interviews and focus groups [7]. Questions of the interview guide were refined during an international research meeting with researchers of the IMPACT project (Additional file 1). To test the interview guide, at least two pilot interviews were conducted per country. Interview and focus groups were recorded with either written or verbal consent of the participants and transcribed verbatim using an agreed transcription format. All focus group interviews were led by experienced moderators.

### Data analysis

Analysis started after the first interview. In each country, researchers (see Additional file 2) condensed data and suggested codes closely related to the text fragments by using a constant comparative method [15]. To control for subjectivity, two researchers per country independently coded the data. Software for the analysis of qualitative data (such as Atlas.ti and MAXQDA) was used to facilitate the coding process. Codes and associated text fragments were translated into English and shared between the researchers. At an international IMPACT research meeting, the interview guide was evaluated and adapted where necessary and a consensus codebook was made. Next, this codebook was used by the researchers for the analysis of the remaining interview and focus group data: two researchers in each country discussed the codes until consensus was reached. When no consensus could be reached, a third researcher was consulted. Categories were derived from the codes and discussed between the researchers in the five countries via email and Skype meetings. Per country, a report was produced, summarizing the barriers and facilitators to improve the organization of palliative care in the respective countries. Two researchers (JvRP and FB) compared all these reports of the individual countries, applying an adapted version of the Grol and Wensing model [16] for understanding change at different levels of health care in order to organise the barriers and facilitators into categories. The adapted model consisted of five themes instead of six: innovation, individual professional level, group dynamics, organizational context and local political-economic context. Categories were fed back and checked with researchers from each country.

### Ethical considerations

The Medical Ethics Committee of the district Arnhem-Nijmegen has declared that this study does not fall within the remit of the Medical Research Involving Human Subjects Act (WMO) (registration number 2012/075). This means that this study can be carried out without an approval by an accredited medical ethics committee.

## Results

In total, 40 professionals were interviewed and another 59 participated in 10 focus group interviews (Table 1). They were spread evenly in all major services providing palliative care, including hospitals, hospices, nursing homes and primary care facilities.

**Table 1:** **Interviewee characteristics**

	**EN**	**DE**	**IT**	**NO**	**NL**
# Interviews	4	2	11	10^a^	9^a^
Male	-	-	4	1	5
Female	4	2	7	11	6
Physicians	3	-	8	3	5
Nurses	1	1	2	3	5
Social worker	-	1	-	-	-
Psychologist	-	-	1	-	-
Managers	-	-	-	6	1
# Focus groups	1	3	2	2	2
Male	-	6	6	1	4
Female	2	9	8	8	15
Physician	-	5	6	-	6
Nurses	2	6	3	9	4
Social worker	-	1	-	-	-
Psychologist	-	-	1	-	1
Manager	-	-	-	-	1
Other	-	3	4	-	7

*EN* England, *DE* Germany, *IT* Italy, *NO* Norway, *NL* The Netherlands.

^a^In both Norway and the Netherlands, two interviews were conducted with two interviewees. The number of interviews (*n* =36) is therefore lower than the actual number of interviewees (*n* =40).

Barriers and facilitators were inductively categorized into 16 categories and organized into themes, using the adapted model for understanding change at different levels of health care [16]. Table 2 provides an overview of the framework with categories, barriers and facilitators, and associated quotes in each theme. Table 3 provides an overview of the categorized barriers and facilitators per country. The themes and categories are summarized below.

**Table 2:** **Themes, categories, codes and associated quotes**

**Themes**	**Categories**	**Codes**	**Associated quotations**
Innovation	Accessibility	Time of training, Availability of education, Frequency of contact	[…] we are trying to organize different moments during the year when all our professionals come here […] to update all together their training program (*psychologist, primary care, Italy*).
	Attractiveness	Method of presentation, (lack of) tailoring, Extrinsic motivation, Extrinsic incentives	Cases were presented, cases from our own organization, cases which really increased motivation of the staff (*manager, hospice, The Netherlands*).
	Usefulness of change	Usefulness, Impact of research, Use of new knowledge	[…] it is important that you will also see the results of what you are doing (*nurse, hospice, The Netherlands*).
Individual professional level	Attitude	Intrinsic interest, Intrinsic motivation, Decision making process	[…] I say ‘interest’, I don’t know - but maybe it is more interest in end of life care or dementia or whatever and that obviously makes life a lot easier when new initiatives and services are available (*nurse, hospice, England*).
			Not all professionals have the proper motivation, time, availability or willingness to involve themselves in something that goes beyond their daily work (*psychologist, primary care service, Italy*).
	Professional skills	Practitioner autonomy, Placing responsibility, Stepwise introduction of new responsibilities	We [physicians] used to administer the chemotherapy. This has now been completely delegated to the nurses. […] the doctor became more an observer. […] the number of patients has increased, so you could not sit there and watch the treatment proceed for 3 hours, so things had to change. The nurses’ competence is much, much more extensive than before (*physician, hospital, Norway*).
	Knowledge	Level of knowledge, Knowledge of palliative care services, (lack of) skills, (lack of) experience	If you only experience 20–25 deaths per year within the entire organisations, it is difficult for the individual nurse to maintain the necessary skills to care for these patients (*physician, nursing home, The Netherlands*).
			Nurses need to know what they can improve before they can improve […] (*physician, nursing home, The Netherlands*).
	Awareness	(lack of) awareness of palliative care	We should make professionals understand that palliative care doesn’t represent the last step […] (*physician, hospice, Italy*).
			Despite all our efforts and education provided, there isn’t a culture about palliative care in everyone yet. For example, it is frustrating when GPs don’t refer their patients to us because they are still conscious (*nurse, hospice, Italy*).
Group dynamics	Team climate	(lack of) group support, Culture of change, Fear and avoidance, Participation	The team doesn’t support each other, […] those who are motivated to change are so few that it is too difficult for them to stand up against those who are against changes (*manager, hospice, The Netherlands*).
			We were trained so traditionally that most of the time, the doctors led the meetings. The others who were present just sat there and answered the questions they were asked, instead of considering themselves as equal members of the team with an active role in the meeting (*manager/nurse, palliative care unit, Norway*).
	Network	Forced network, Knowing other professionals/services, Competition between services	[…] there is an increasing number of services and offers, meaning it is becoming much more complicated […] (*social worker, palliative care unit, Germany*).
			[…] within such a network, people interact who cannot stand each other, but we ask them to do so (*physician, nursing home, The Netherlands*).
	Professional guidance	Role modeling, Mentoring, Feedback	We have a retired GP who is really good, […] who goes out to see the GPs in […] that worked really well, he was well regarded in his role. So, of course, him going back to the GPs, they think that’s marvelous, you know, they respect him (*nurse, hospice, England*).
			With that colleague I took the time to discuss what the possibilities were and showed him what he could improve. This practical contact really made a difference (*physician, hospital, The Netherlands*)
Organizational context	Organizational processes	Physical structures, Managing complexity, Extrinsic interest, Use of technology	[…] we need to work a lot with temporary personnel, which brings along the problem that they cannot take part in meetings of quality circles. This means that it is extremely difficult to implement agreed standards (*head nurse, primary care, Germany*).
	Organizational structures	Structure of organization, Place of care	[…] the hospital itself has changed from being one big building to several big buildings. We used to meet colleagues in the cantina. But now we’re too busy, so we never go to the cantina and if you do, you go to different cantinas, so you don’t meet colleagues like you used to. The lobbying you could do earlier, you can’t do that anymore (*physician, hospital, Norway*).
	Staff	Staff size, Staff turnover, Availability of staff, Hiring new staff, Depletion of other service	[…] sometimes you have to deal with a culture that is very much dependent on the persons working there. If some of these persons leave, it becomes very difficult to maintain innovations (*physician, nursing home, The Netherlands*).
			[…] there are only few people interested in qualifying, choosing this profession is becoming increasingly unattractive […] (*physician, palliative care unit, Germany*).
	Time	Time constraints, Burden of information	[…] so busy with caseload stuff that you haven’t got the time or as much time as you’d like to do that education bit and training (*nurse, hospice, England*).
			[…] you are so busy every day that you don’t find the time to meet people (*physician, palliative care unit, Norway*).
Economic and political context	Financial arrangement	(lack of) resources, Financial aspects, Financial incentives	If you […] need an additional employee […], this will cost money. If I don’t have the money, I won’t have the employee, if I don’t have the manpower for this task, I may put less effort in documentation work. And if then someone comes and says: The documentation is not appropriate … Well, what would be the reason? Lack of resources. I think, this is where one shoots oneself in the foot (*physician, hospital, Germany*).
			Other medical areas […] receive funding from large (pharmaceutical) industries. Palliative care doesn’t have that kind of support (*general practitioner, The Netherlands*).
	Regulations	Availability of (existing) guidelines/rules, Formalization of change	Everything, […] yes, it needs to be in concordance with the principles of the whole organization (*director, nursing home, Germany*).
			When palliative care was introduced, the national organisation was primarily focused to improve cure within the hospital and not care within primary care (*general practitioner, The Netherlands*).

Each citation is supplemented with the type of profession, setting and country of the professional involved.

**Table 3:** **Overview of barriers and facilitators of strategies to improve the quality of palliative care**

**Themes**	**Facilitators**					**Barriers** ^**a**^				
	**EN**	**DE**	**IT**	**NO**	**NL**	**EN**	**DE**	**IT**	**NO**	**NL**
Innovation		Usefulness	Accessibility, attractiveness, usefulness	Accessibility, attractiveness, usefulness	Accessibility, attractiveness, usefulness		Usefulness	Attractiveness	Attractiveness	Accessibility, attractiveness
Individual professional level	Attitude, professional skills	Attitude, professional skills, knowledge, awareness	Attitude, professional skills	Attitude, professional skills, knowledge	Attitude	Attitude	Attitude, knowledge, awareness	Attitude, knowledge		Attitude, awareness
Group dynamics	Professional guidance	Team climate	Team climate, professional guidance, network	Network	Professional guidance, network		Team climate	Team climate, network	Team climate	Team climate, network
Organization-al context		Organizational processes, organizational structure		Staff	Organizational processes	Time, staff		Time	Time, staff, organizational structure	Time, staff, organizational structure
Economic and political context	Financial arrangement, regulations		Regulations	Financial arrangement, regulations	Regulations	Financial arrangement, regulations	Financial arrangement	Financial arrangement, regulations	Financial arrangement, regulations	Financial arrangement, regulations

*EN* England, *DE* Germany, *IT* Italy, *NO* Norway, *NL* The Netherlands.

^a^For the readability of the table, the ‘lack of’ has been left out in the description of barriers. However, each barrier should be read as if there is a lack of it, e.g. lack of attractiveness, lack of time, etc.

### Innovation

Three categories emerged: (1) accessibility of improvement strategies, (2) attractiveness of improvement strategies and (3) usefulness of change.

#### Accessibility of improvement strategies

Interviewees in Italy, Norway and the Netherlands stated that it is important that improvement strategies (such as education) are arranged in a way that as many professionals of the same team as possible can participate. Professionals in the Netherlands also stated that they perceived restricted access to the improvement strategies as a barrier, for example when the training frequency was low.

#### Attractiveness of improvement strategies

Professionals in Italy, Norway and the Netherlands considered the attractiveness of improvement strategies important. They stated that the perceived attractiveness of an improvement strategy increases when it is tailored to the needs of the service in question. Interactive educational methods and enthusiasm and motivation of those responsible were considered important contributors to the attractiveness of an improvement strategy. The attractiveness of quality improvement projects was also facilitated by certifying participants for their participation, e.g. for having received education.

#### Usefulness of change

Perceived usefulness of quality improvement projects was mentioned as an important facilitator by professionals in all countries except England. Professionals were, for example, more motivated to collect data to measure the quality improvement or use specific tools when these activities benefitted their own clinical practice.

### Individual professional level

Four categories were related to this theme: (1) professional skills, (2) attitude of professionals, (3) knowledge and (4) awareness of palliative care.

#### Professional skills

The introduction of new professional skills, which are expected to become part of behavioural routines, was mentioned as an important facilitator for the success of quality improvement projects by professionals in all countries but the Netherlands. A Norwegian professional, for example, clarified that the education nurses receive now is much more extensive than it used to be, enabling task delegation from physician to nurse.

#### Attitude of professionals

A positive attitude of professionals regarding improvements was considered an important factor for the success of quality improvement projects by professionals in all countries. Participation in staff training is, for example, facilitated when staff members are motivated and have an interest in the topic. Professionals in England, Germany, Italy and the Netherlands also stated that reluctance of professionals, as well as of organizations, to participate contributes to erroneous beliefs about palliative care, adherence to obsolete routines and may be due to the pressure on the organization to participate in many improvement projects.

#### Knowledge and awareness of palliative care

Knowledge and awareness of palliative care were mentioned by professionals in Germany, Italy, Norway and the Netherlands. Professionals in Italy, for example, described that there is a general lack of awareness about palliative care, and professionals in the Netherlands stated that their managers considered palliative care unimportant, because they were not aware of what palliative care actually is. However, they also stated that by improving their knowledge, their motivation and interest to change increased.

### Group dynamics

Three categories emerged: (1) professional guidance, (2) team climate and (3) participation in a network.

#### Professional guidance

Professionals in England, Norway and the Netherlands mentioned that professionally guiding people in their practice and performance, such as role modelling and mentoring, has a positive effect on their performance and was therefore perceived as facilitator for many change strategies.

#### Team climate

The importance of a positive team climate was mentioned by professionals in Germany, Italy, Norway and the Netherlands. Top-down implementation, for example, was not considered to be effective. A well-balanced team with the involvement of staff in decision making regarding implementation of changes was—in general—considered more effective. Also, several interviewees reported that some of their colleagues were reluctant to change, which decreased the motivation of the team to change. Existing norms and values which were difficult to change were the underlying reasons for this reluctance. In the Netherlands, being involved in too many improvement projects at the same time was perceived as an exhausting factor for the team.

#### Participation in a network

Being part of a palliative care network was an important facilitator in Italy, Norway and the Netherlands. Professionals involved in a network mentioned that it helped them to know other professionals in their organization, making it easier to initiate quality improvement projects. However, participation in networks was also perceived as a barrier since issues of collaboration and communication took up too much time at the expense of other improvement strategies (in Norway), involved cooperation with people that sometimes was perceived as difficult (in the Netherlands) and resulted in competition between services to get funded (also in the Netherlands).

### Organizational context

There are four categories in this theme: (1) organization of care processes, (2) organizational structure, (3) availability of staff and (4) availability of time to implement improvement strategies.

#### Organization of care processes

Professionals in England, Germany, Italy and the Netherlands stated that it is easier to implement changes when they are in harmony with the general principles of care of their institution. It was also mentioned that having access ‘to the right people’ (e.g. management) made it easier to initiate quality improvement projects.

#### Organizational structure

The infrastructure of a service (e.g. physical and spatial structure of the building where it is located but also the hierarchical structure of the organization) was mentioned as a barrier by German, Norwegian and Dutch professionals. For example, spending too much time travelling within or between buildings and a shortage of facilities such as rooms for educational activities were perceived as barriers.

#### Availability of staff

Staff shortages were especially experienced in England, Norway and the Netherlands. Not having enough staff to allow training without disruption of clinical care created difficulties in improving the organization of palliative care. High staff turnover results in a never-ending need for training while the services do not have the resources to supply this demand, resulting in professionals having little time to update themselves professionally. However, in services with extra financial resources, hiring extra staff to work shifts for the permanent employees, facilitated their participation in educational sessions. Norwegian professionals commented that recruiting experienced personnel is a rather quick way of increasing palliative care expertise amongst staff. However, they also stipulated that this is not always a good solution since it will deplete other services.

#### Availability of time

Lack of available time to participate in improvement projects was perceived as an important barrier in all countries, but mentioned from two perspectives. Firstly, professionals are faced with extreme time constraints in their clinical work, which limits their availability for training, participation in improvement projects and keeping up to date with new knowledge. Secondly, quality improvement projects may require strategies that take a considerable amount of time to implement, which consequently puts an additional burden on the professionals and organization. In Germany, lack of time was also considered a facilitator if the innovation would result in saved time, as it helped to focus on the benefits of change.

### Local political-economic context

Two categories emerged: (1) financial arrangements and (2) effective organizational regulations.

#### Financial arrangements

In all countries, interviewees mentioned that extrinsic financial incentives are crucial for the effectiveness of implementation strategies designed to promote service improvement. For example, there has been a lack of recruitment in services in England and Norway due to financial barriers, because specialist staff were considered too expensive. Financial constraints also resulted in truncation of quality improvement projects, limiting their effect.

#### Organizational regulations

Interviewees in all countries except for Germany reported that they experienced existing regulations on a national, regional and local level both as a facilitator as well as a barrier to changing practice. Professionals considered them a facilitator because clear organizational regulations facilitated participation in quality improvement projects and ensured the quality of care. However, interviewees in the Netherlands perceived them as a barrier because new regulations caused them a lot of extra work. In Italy, some professionals mentioned that the fixed number of certain staff in nursing homes (e.g. not enough staff in relation to the number of patients) negatively affected their work and consequently the success of improvement projects. Professionals in England and Norway also mentioned that policies and guidelines in place to protect patient information can, for example, also limit the use of innovative quality improvement strategies, such as the use of an electronic patient file.

## Discussion

This study identified barriers and facilitators to improve the organization of palliative care. They could be arranged in five themes as described by Grol, being the innovation itself, the individual professional level, group dynamics, the organizational context and the local political-economic context [16]. All themes appeared to be related to structures and processes of care, as described in the Donabedian Model [17]. Although the barriers and facilitators differ in scope, context, strength and provenance, most of them were shared by professionals from different European countries. However, when comparing barriers and facilitators cross-nationally, differences in the provision of palliative care should be considered. For example, the national health-care system and organization of palliative care differs in the five participating countries. As shown elsewhere, all five countries have legislation about palliative care [18,19]. Despite broadly similar legal frameworks, access to palliative care services differs between countries [20]. There are, for example, cultural barriers in Italian society that refrain patients from receiving adequate and timely palliative care [18,21]. Although such barriers were not reported by professionals in the other countries, some of them experienced a lack of awareness, but then primarily caused by a lack of knowledge about palliative care of their superiors. Furthermore, the availability of palliative care services also differed between countries. However, even though hospices are not available in Norway and Italy does not have palliative care units in hospitals [18], patients receive palliative care in other types of services, whereby most services provide palliative care in agreement with the World Health Organization’s definition of palliative care [18,22].

Differences were found not only between countries but also within countries, such as regional or setting-specific regulations. Sometimes, the same aspect appeared to be a facilitator in one service and a barrier in another. In the Netherlands, for example, networking was considered a facilitator in one service as it resulted in knowing other professionals, but a barrier in another service, as economic regulation caused competition between services.

Despite these national differences, it appeared that similar barriers and facilitators regarding the organization of palliative care existed in different countries. For example, team climate was mentioned as an influential factor by professionals in all countries but England, and organizational processes were mentioned by professionals in Germany as well as in the Netherlands. Financial resources and a positive attitude to change were mentioned by professionals in all countries, suggesting that, for example, sufficient funding and the motivation of staff are a prerequisite to change. Facilitators that were mentioned as a barrier when absent (e.g. attractiveness of improvement strategies) could also be considered as an essential requirement to change.

Several facilitators identified in this study are comparable to those found in other studies in health care, like flexibility of timing educational sessions [23], enthusiastic and active initiators [24] and intrinsic motivation of the team members [25]. Comparable barriers are lack of awareness [25], lack of training and guidance [26], fear of change [26], time constraints [21], staff shortages [21,27], lack of funding [21,27] and lack of adherence to guidelines [28]. The factors identified in our study are therefore not unique to palliative care, but it appears that there are similarities between the organization of care in different services and countries. Even though the factors may not be unique, the combination of them is relevant because of the complexity of palliative care [21]. Patients in need of palliative care, for example, have multiple problems and symptoms resulting in changing (and often increasing) needs for treatment and support [4]. Consequently, they receive care from a variety of professionals in different types of services [1,6]. This requires multidisciplinary teamwork and a good division of tasks and responsibilities [1,6]. However, the multidisciplinary approach in palliative care is also what makes it more difficult to change the organization of palliative care [5]. In our study, a Norwegian interviewee, for example, pointed out that because staff were trained so traditionally, physicians led the team meetings, while the other staff present did not participate. West et al. described ‘participation safety’ as defined by the extent to which a team participates in making decisions and whether team members feel psychologically safe in proposing new ideas, as a factor that can influence teamwork [29]. In this case, ‘participation safety’ was not possible because of the attitude of the nurses and the social pressure of the physicians. Together with self-efficacy, attitude and social influence are the main determinants of the ASE Model (Attitude, Social-influence and self-Efficacy) [30], which in itself is derived from the theory of planned behaviour [31]. Knowing these determinants can facilitate adaptation of improvement strategies in order to achieve the planned behaviour of the nurses. However, only few implementation models translating the results of research into clinical routines recommend to perform a detailed analysis of barriers and facilitators before starting the intervention [7,32]. For example, the widely used framework for the development and evaluation of complex interventions to improve health of the UK Medical Research Council does not consider such uncertainties until the pilot testing of the intervention [33]. The PDSA cycle (Plan-Do-Study-Act) does not even explicitly mention a barrier and facilitator analysis [34]. Although most implementation studies refer to one of these models, only few studies actually perform a barrier and facilitator analysis before starting to implement an intervention [35]. The barriers and facilitators identified in this study will be used in the IMPACT project to tailor country and setting-specific intervention strategies to improve the organization of palliative care in 40 services across Europe.

### Strengths and limitations

A strength of this study is that it is a large study, conducted with individual and focus group interviews in five European countries. Professionals working in the field of palliative care in hospitals, hospices, primary care settings and nursing homes were included. The results of our study can therefore be used in a variety of services, addressing not only patients with cancer but also patients with dementia in need of palliative care. A limitation of this study is that the interviews were conducted in five different languages. Although a common format was used for transcription and translation and meanings were reviewed in consensus meetings, different native languages may have caused differences in interpretation. Second, the aim of this study was to identify barriers and facilitators to improve the organization of palliative care in different European countries. It is therefore a limitation that factor shaping strategies for service changes may be system-specific and not identified in our sample.

## Conclusion

This study identified barriers and facilitators to organizational change in palliative care. Some of these barriers and facilitators were experienced by professionals in almost all countries and are therefore prerequisites to change. In order to promote successful implementation of change, it is important to tailor an organizational improvement to the needs of individuals and organizations. Understanding the barriers to and facilitators of change is essential for such tailoring.

## Additional files

Additional file 1:
**Interview guide.** An interview guide was used for the semi-structured individual interviews and focus group interviews. Additional file 2:
**Researcher information.** Professional background and involvement in data collection and analysis of researchers that participated in this study.
