# Supplementary material for: Improving the organization of palliative care: identification of barriers and facilitators in five European countries
Source: Implement Sci. 2014 Oct 16;9:130. doi: 10.1186/s13012-014-0130-z (PMC4203898; doi:10.1186/s13012-014-0130-z)
Supplement: Additional file 2: — Researcher information. Professional background and involvement in data collection and analysis of researchers that participated in this study. [file 13012_2014_130_MOESM2_ESM.docx]

**Researcher information**

| **Country** | **Researcher** | **Professional Background** |
| --- | --- | --- |
| England | ND | Researcher (PhD student) at University College London, with a professional background in psychology. Responsible for the interviews and analysis in England. |
| Germany | BJ | Senior researcher at the Universities of Bonn and Göttingen, with a professional background in philosophy. Responsible for the interviews and analysis in Germany. |
| Italy | EM | Researcher at the University of Bologna, with a professional background in psychology. Responsible for the interviews and analysis in Italy. |
| Norway | RS | Researcher (PhD student) at the Norwegian University of Science and Technology, with a professional background in sociology. Responsible for the interviews and analysis in Norway. |
| The Netherlands | JvRP | Researcher (PhD student) at the Radboud university medical center, with a professional background in health sciences and nursing. Responsible for the interviews and analysis in the Netherlands, and for the comparison of data between countries. |
